# Lipid Metabolic Process Involved in Oocyte Maturation During Folliculogenesis

**DOI:** 10.3389/fcell.2022.806890

**Published:** 2022-03-31

**Authors:** Tao Liu, Jiangxue Qu, Mengyuan Tian, Rui Yang, Xueling Song, Rong Li, Jie Yan, Jie Qiao

**Affiliations:** ^1^ Center for Reproductive Medicine, Department of Obstetrics and Gynecology, Peking University Third Hospital, Beijing, China; ^2^ National Clinical Research Center for Obstetrics and Gynecology, Beijing, China; ^3^ Key Laboratory of Assisted Reproduction, Ministry of Education, Beijing, China; ^4^ Beijing Key Laboratory of Reproductive Endocrinology and Assisted Reproduction, Beijing, China; ^5^ Research Units of Comprehensive Diagnosis and Treatment of Oocyte Maturation Arrest, Chinese Academy of Medical Sciences, Beijing, China

**Keywords:** lipid metabolism, meiotic resumption, folliculogenesis, β-oxidation, lipoprotein, cholesterol

## Abstract

Oocyte maturation is a complex and dynamic process regulated by the coordination of ovarian cells and numerous extraovarian signals. From mammal studies, it is learnt that lipid metabolism provides sufficient energy for morphological and cellular events during folliculogenesis, and numerous lipid metabolites, including cholesterol, lipoproteins, and 14-demethyl-14-dehydrolanosterol, act as steroid hormone precursors and meiotic resumption regulators. Endogenous and exogenous signals, such as gonadotropins, insulin, and cortisol, are the upstream regulators in follicular lipid metabolic homeostasis, forming a complex and dynamic network in which the key factor or pathway that plays the central role is still a mystery. Though lipid metabolites are indispensable, long-term exposure to a high-fat environment will induce irreversible damage to follicular cells and oocyte meiosis. This review specifically describes the transcriptional expression patterns of several lipid metabolism–related genes in human oocytes and granulosa cells during folliculogenesis, illustrating the spatiotemporal lipid metabolic changes in follicles and the role of lipid metabolism in female reproductive capacity. This study aims to elaborate the impact of lipid metabolism on folliculogenesis, thus providing guidance for improving the fertility of obese women and the clinical outcome of assisted reproduction.

## Introduction

An ovarian follicle is the basic reproductive unit of the mammalian ovary and consists of oocyte and follicular somatic cells, including pre-granulosa cells, granulosa cells, theca cells, vascular endothelial cells, and immune cells. With the development and maturation of the hypothalamic–pituitary–ovarian (HPO) axis after puberty, primordial follicles develop sequentially through primary, secondary, and antral stages before ovulation under the control of growth factors, hormones, and other ovarian internal and external factors (Dong et al., 1996; Durlinger et al., 2002; Sekulovski et al., 2020). Ovulation occurs only in less than 1% of follicles, whereas the remaining 99% undergo atretic degeneration.

The follicle microenvironment is a multidimensional biological system of complex components including oocytes, granulosa cells, theca cells, and immune cells and signaling molecules, such as steroid hormones, growth factors, and extracellular matrix constituents, which are necessary for folliculogenesis and oocyte maturation (Shea et al., 2014; Dumesic et al., 2015). The maintenance of the follicular microenvironment homeostasis depends on normal metabolism and accurate communication in and between cells (Dumesic et al., 2015).

Lipid metabolites are important in cell signaling and metabolic processes. For example, fatty acids are the energy providers in oocytes (Dunning et al., 2014), and cholesterol is the precursor of steroid hormones synthesized in granulosa cells and theca cells. Some lipid metabolites are also important regulators of oocyte meiosis and maturation (Marín Bivens et al., 2004a; Ben-Ami et al., 2006). On the other hand, excessive lipid accumulation will cause serious damage to ovarian reproductive function by inducing ovarian oxidative stress and inflammation (Gu et al., 2015; Cardozo et al., 2016). Based on mammal studies, this review will summarize the role of lipid metabolism in folliculogenesis and oocyte maturation and discuss the potential application of lipids in improving the oocyte *in vitro* maturation (IVM) systems.

## Triglyceride Breakdown

Triglycerides are an important storage form of energy, which mainly exist in the form of lipid droplets. Though the content of lipid droplets varies greatly among different species and developmental stages, mammalian oocytes are generally rich in lipids to ensure sufficient energy supply for meiosis (Genicot et al., 2005; Ariu et al., 2016; Abazarikia et al., 2020).

**FIGURE 1:**
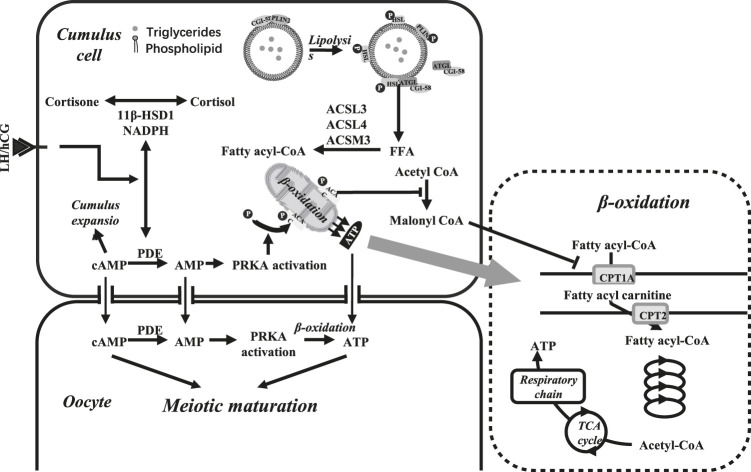
Triglyceride breakdown and fatty acid oxidation in oocyte and cumulus cells. Triglyceride breakdown: In the presence of hormones or fatty acids, phosphorylated PLIN loses the protection on triglycerides and the binding capacity on CGI-58, thus activating HSL and ATGL and promoting lipolysis. Fatty acid oxidation: In the presence of LH or hCG, 11β-HSD1 in the cumulus cells of preovulatory follicles catalyzes corticosterone into active cortisol. Activated PDE promotes the decomposition of cAMP, thereby promoting cumulus expansion. PRKA is activated by the AMP phosphorylate and inactive ACAC, which then downregulate the conversion of acetyl CoA to malonyl CoA and promote *β*-oxidation. cAMP and AMP can also be transported into oocytes *via* gap-junction, thus promoting meiotic maturation together with the ATP from cumulus cells and *β*-oxidation in oocytes. The pathway in the dotted line box showed the process of β-oxidation.

Triglyceride catabolism requires several lipolysis-related proteins, including perilipin (PLIN), comparative gene identification 58 (CGI-58), hormone sensitive lipase (HSL), and adipose triglyceride lipase (ATGL), in which PLIN plays a central role in lipid homeostasis by coating on the surface of lipid droplets (MacPherson and Peters, 2015). In the presence of hormones or fatty acids, PLIN and HSL are phosphorylated through the G protein–cyclic adenosine monophosphate (cAMP)-protein kinase A (PKA) signaling pathway. Losing the barrier protection of PLIN, triglycerides are decomposed to fatty acids by phosphorylated HSL that accumulate around lipid droplets (MacPherson and Peters, 2015) (Figure 1). Nonphosphorylated PLIN can bind directly with CGI-58, while the binding capacity of phosphorylated PLIN is largely reduced, making CGI-58 translocate to the cytoplasm and activate ATGL to promote lipolysis (MacPherson and Peters, 2015) (Figure 1).

The expression levels of lipolysis-related factors in oocytes at different stages have been reported by oocyte IVM experiments in many species (bovine, porcine, and mice) (Yang et al., 2010; Sastre et al., 2014; Zhang et al., 2014; Xu et al., 2018). Though PLIN plays a bidirectional role in regulating triglyceride metabolism, PLIN2 inhibits lipolysis and promotes lipid synthesis, and PLIN3 contributes to lipid droplet formation on the whole (Sztalryd and Kimmel, 2014). The expression level of PLIN2 in the *in vitro* matured oocytes was comparable or lower than that of oocytes at the GV stage, and the expression of PLIN3 was significantly decreased (Sastre et al., 2014; Zhang et al., 2014; Xu et al., 2018). Different from the expression in oocytes, PLIN2 and PLIN3 were expressed less in cumulus cells from mature oocytes compared to those from immature oocytes (Xu et al., 2018; Feuerstein et al., 2012; Sanchez-Lazo et al., 2014)). Although studies on the gene expression of *in vivo* matured oocytes are limited, the *in vitro* studies suggest that lipolysis was increasingly active as oocytes mature.

Hormones in granulosa cells or blood circulation can also regulate lipid metabolism in follicles. 11β-hydroxysteroid dehydrogenase type 1 (11β-HSD1) in preovulatory follicular cumulus cells catalyzes corticosterone into active cortisol in the presence of luteinizing hormone (LH) or human chorionic gonadotropin (hCG) (Yong et al., 2000). Cortisol in follicular fluid is negatively correlated with the quantity of lipid droplets in cumulus cells, while it is positively correlated with the number of mature oocytes (Simerman et al., 2015). In addition, better *in vitro* fertilization (IVF) outcomes, including successful oocyte fertilization, embryo implantation, and clinical pregnancy, were associated with higher concentration of cortisol and cortisol: corticosterone ratio in follicular fluid (Keay et al., 2002; Lewicka et al., 2003). Follicular cortisol may interact with or increase the expression of HSL in cumulus cells and oocytes (Stimson et al., 2017), promoting the breakdown of triglycerides into free fatty acids (Simerman et al., 2015). Therefore, lipolysis controlled by the transformation of corticosterone into cortisol may be an important mechanism regulating oocyte meiotic resumption (Figure 1).

## Fatty Acid Oxidation

Fatty acids obtained from the breakdown of triglycerides are activated to acyl coenzyme A (CoA) in the endoplasmic reticulum and mitochondrial outer membrane (Figure 1). Acyl CoA is transported into the mitochondria by carnitine palmitoyltransferase (CPT1 and CPT2). It is then oxidized to produce one acetyl CoA, one acyl CoA, and one NADH^+^, H^+^, and FADH_2_ after four steps of dehydrogenation, hydration, oxidation, and thiolysis (Figure 1). Acetyl CoA is converted into ATP after entering the tricarboxylic acid cycle (TCA cycle) (Figure 1). Comprising a long hydrocarbon chain, fatty acids can produce multiple acetyl CoAs in β-oxidation, thus yielding more energy than glucose with the same weight or number of carbons.

β-oxidation and meiotic resumption are closely related and mutually reinforced, in which adenosine monophosphate (AMP) and adenosine monophosphate–activated protein kinase (PRKA) play critical roles. cAMP is a recognized meiotic inhibitor. It can activate protein kinase A (PKA), the activator of various intranuclear kinases and inhibitor of cyclin-dependent kinases, thus keeping the maturation promoting factor (MPF) inactive. Induced by physiological or pharmacological gonadotrophins (e.g., LH or hCG), phosphodiesterase (PDE) in cumulus cells is activated and stimulates the decomposition of cAMP, thereby relieving its negative effect on MPF activity and promoting meiotic resumption (Gilchrist et al., 2016) (Figure 1). Malonyl CoA, the precursor for fatty acid synthesis, can inhibit the synthesis and promote oxidative decomposition of fatty acids by inactivating CPT1. Acetyl CoA carboxylase (ACAC) mediates the conversion of acetyl CoA to malonyl CoA (Hardie and Pan, 2002) (Table 1). cAMP decomposition is accompanied by the synthesis of AMP, an activator of PRKA (Hardie, 2011; Valsangkar and Downs, 2013) which phosphorylates and inactivates ACAC in the presence of LH or hCG (Yong et al., 2000; Simerman et al., 2015). This may be the main mechanism of PRKA-mediated upregulation of β-oxidation during meiotic resumption (Simerman et al., 2015). cAMP, AMP, and ATP in cumulus cells can also be transported into oocytes *via* gap junction (Gilchrist et al., 2004; Richani et al., 2019). cAMP and ATP function through signal transduction and energy supply, respectively, promoting cumulus expansion and oocyte meiotic maturation. In addition, as a synthetic substrate of ADP and decomposition product of ATP, an increase in the AMP level can also direct the nucleoside phosphate conversion process to generate more ATP so that oocytes can release a large amount of energy in a short time.

**TABLE 1:** Summary of the association between lipid metabolism pathways and oocyte maturation or fertility.

Pathway	Key enzyme	Method	Influence on oocyte maturation	Phenotype
Lipolysis and β-oxidation	CPT	Adding inhibitor of CPT in the IVM medium	Suppress (Valsangkar and Downs (2013))	—
	—	Adding activator of CPT in the IVM medium	Promote (Valsangkar and Downs (2013))	—
Fatty acid synthesis	FASN	Adding inhibitor of FASN in the IVM medium	Promote (Downs et al. (2009))	—
	ACAC	Adding inhibitor of ACAC in the IVM medium	Promote (Valsangkar and Downs (2015))	—
	—	Adding activator of ACAC in the IVM medium	Suppress (Valsangkar and Downs (2015))	—
Lipoprotein transport	SCARB1	*Scarb1* ^−/−^ mice	—	Infertile (Trigatti et al. (1999), Miettinen et al. (2001))
	LDLR	*Ldlr* ^−/−^ mice	—	Fertile but with decreased ovarian reserve (Review in Fujimoto VY, (2010))
Lipoprotein synthesis and remodeling	ABCA1	Abca1^−/−^ mice	—	Fertile but with embryopathy (Review in Fujimoto VY, (2010))
	LCAT	Lcat−/− mice	—	Fertile (Review in Fujimoto VY, (2010))
	LXR	LXR^−/−^ mice	—	Fertile but with decreased ovarian reserve (Review in Fujimoto VY, (2010))

## Fatty Acid Synthesis

The liver is the major site for fatty acid synthesis, of which the first step is the conversion of acetyl CoA to malonyl CoA (de Carvalho and Caramujo, 2018). In the presence of NADPH and fatty acid synthase, the carbon chain is elongated to 16 (palmitic acid) after seven cycles of condensation, reduction, dehydration, and reduction. Palmitic acid is processed and elongated in the hepatocellular endoplasmic reticulum and mitochondria to produce fatty acids with longer carbon chains (>16) (Kawano and Cohen, 2013).

Fatty acid synthesis is important in storing excess energy for later use. However, the role of this process in meiotic resumption and oocyte maturation is very limited. Additional cerulenin, a natural inhibitor of fatty acid synthase (FASN), in the *in vitro* maturation (IVM) medium can promote meiotic resumption in COCs but not in denuded oocytes (Downs et al., 2009) (Table1). These results indicate that the inhibition of fatty acid synthesis in granulosa cells can promote oocyte maturation, while the inhibition of fatty acid synthesis in oocytes cannot promote oocyte maturation, which implies the absence or limited fatty acid synthesis activity in oocytes. C75, a promoter of fatty acid oxidation and inhibitor of fatty acid synthesis, has a stronger enhancing effect on meiotic resumption than cerulenin and can promote meiotic resumption in both COCs and denuded oocytes. Etomoxir, an inhibitor of fatty acid oxidation that acts through inhibiting CPT1, can block the effects of C75 on meiotic resumption (Downs et al., 2009). These *in vitro* experiments indicate that the role of fatty acid oxidation is more crucial than that of fatty acid synthesis in oocyte meiotic resumption.

## Lipoprotein Transport

Human lipoproteins are classified into four types according to their density: chylomicrons (CMs), very low density lipoprotein (VLDL), low-density lipoprotein (LDL), high-density lipoprotein (HDL), and lipoprotein (a) [Lp (a)], each having its specific protein and cholesterol composition, synthesis, transport and metabolic pathways, and biological functions. Intestinal mucosa–produced CMs transport exogenous triglycerides and cholesterol digested from food. VLDL is produced by hepatocytes and transports endogenous triglycerides and cholesterol. VLDL is converted into LDL by the catalysis of lipoprotein lipase (LPL) and hepatic triglyceride lipase (HTGL), transporting endogenous cholesterol in the body. Neonatal HDL is synthesized both in the liver and intestine, which absorbs excess cholesterol from extrahepatic tissues and transports them to the liver, thus removing cholesterol from the body. Previous studies discussed how lipoproteins affect female reproductive function by interfering with the expression and activity of lipoprotein receptors and determined whether or not a lipoprotein is involved in tissue-specific lipid transport.

Scavenger receptor class B type I (SCARB1) mediates the selective uptake of cholesteryl esters from HDL, and its critical effects on female reproduction have been confirmed in several studies. Though having normal ovarian morphology and ovulation rate, *Scarb1*-deficient mice were infertile with accumulated cholesterol in oocytes (Miettinen et al., 2001; Trigatti et al., 1999) (Table 1). Lipid metabolism disorders including a high plasma cholesterol level and high nonesterified cholesterol level in the extraovarian environment account for the infertility phenotype of *Scarb1*-deficient (*Scarb1* ^−/−^) mice. When altering the structure or quantity of abnormal HDL by silencing the *ApoA1* gene or lowering cholesterol levels in blood by feeding probucol, the fertility of *Scarb1*-deficient mice was partially or completely restored (Miettinen et al., 2001; Quiroz et al., 2020). Human plasma cholesteryl ester transfer protein (CETP) transfers cholesteryl esters from HDL and LDL to VLDL, which in turn binds hepatic LDL receptor (LDLR) or VLDL receptor (VLDLR) to transport cholesterol from peripheral tissues to the liver (reverse cholesterol transport). Wild-type mice express no CETP. *CETP* transgenic *Scarb1*-deficient mice showed partially restored HDL particle size. However, the plasma cholesterol level was not restored, and symptoms of several disorders, including reticulocytosis, impaired platelet aggregation, and female infertility, could not be relieved (Hildebrand et al., 2010).

Abnormal oocyte meiosis and activation induced by excess cholesterol directly cause infertility of *Scarb1*-deficient mice. Oocytes of *Scarb1*-deficient mice arrested at the metaphase II stage were spontaneously activated without the sperm *in vivo* and further progressed to pronuclear, MIII, and anaphase/telophase III stages (Yesilaltay et al., 2014). When loaded with excess cholesterol *in vitro*, oocytes from healthy wild-type mice could be spontaneously activated with MPF reduction, mitogen-activated protein kinase (MAPK) activity, and Ca^2+^ concentration oscillations (Yesilaltay et al., 2014). Therefore, excess cholesterol may be the underlying cause of how hypercholesterolemia causes impaired fertility in *Scarb1*-deficient mice.

Based on the association between infertility and excess cholesterol in blood and oocytes, another question was raised: How does hypercholesterolemia cause cholesterol overload in oocytes? Some potential hypotheses of the problem are discussed as follows:

1) ATP binding cassette transporter A1 (ABCA1) mediates the efflux of intracellular free cholesterol to lipid-poor apolipoprotein A–I (apoA–I) and is expressed in mouse oocytes (Quiroz et al., 2020). *Scarb1* knockout–induced hypercholesterolemia may increase cholesterol levels in follicular fluid, which inhibit ABCA1 activity and result in overloaded cholesterol in oocytes (Quiroz et al., 2020; Chang et al., 2017).
2) High cholesterol in follicular fluid enlarges the cholesterol concentration gap among internal and external oocytes and granulosa cells, thus enhancing cholesterol influx and increasing cholesterol levels (Yesilaltay et al., 2014). This hypothesis is supported by the finding that cholesterol in red blood cells and platelets was transported from extracellular HDL into cells of *Scarb1*-deficient mice by simple diffusion of nonesterified cholesterol (Holm et al., 2002).
3) SCARB1 mediates not only the selective uptake of cholesterol but also the partial efflux of cholesterol to immature HDL particles (Fujimoto et al., 2010). Therefore, the cholesterol level in oocytes of SCARB1 knockout mice may also be affected by this abnormal process.

The association between circulating high cholesterol and excess cholesterol in oocytes remains a mystery. Further investigation on this issue is needed to help clarify the mechanism of follicular cholesterol homeostasis maintenance.

The uptake of LDL is dependent on the LDLR-mediated endocytosis (Go and Mani, 2012). Coated pits can form when ApoB-100, a protein component of LDL, binds to the ligand binding domain of LDLR. As a result, coated vesicles can bud inward and eventually dissociate from the membrane (Go and Mani, 2012). After the fusion of the endosome and endocytic vesicles with the LDL–LDLR complex, LDLR will return to the plasma membrane for recycling and LDL will be sent to the lysosome for hydrolysis by lysosomal acid lipase type A, providing cholesterol, fatty acids, and other phospholipids (Go and Mani, 2012). *Ldlr*-deficient mice had lower ovarian lipid, progesterone, and estrogen levels; decreased follicle count; and increased atretic follicles (Table 1). The litter frequency was not changed, while the litter size was reduced significantly (Chang et al., 2017; Guo et al., 2015). *Ldlr*-deficient mice were fertile, indicating that *Ldlr* has limited effect on female fertility than *Scarb1*. In addition to HDL, SCARB1 also mediates the selective uptake of cholesterol esters in LDL, VLDL, phospholipids, and others, making the loss of function of *Scarb1* significantly impact lipid concentration and reproductive function (Huang et al., 2019).

## Lipoprotein Synthesis and Remodeling

Though HDL particles in follicular fluid mainly come from blood circulation, large differences are found between follicular fluid and plasma HDL components. HDL in human follicular fluid contains less cholesterol, more phospholipids, and increased ApoA-4/ApoA-1 levels when compared to plasma (Jaspard et al., 1997). Lipoprotein remodeling and selective filtration by the blood follicle barrier are thought to be the main causes for the differences (Fujimoto et al., 2010). The blood follicle barrier contains vascular endothelial cells, subendothelial basement membrane, theca interna, follicular basement membrane, and mural granulosa cells, providing a physical and biological selective filtration for molecules (Shalgi et al., 1973). Previous studies showed that molecules with a weight under 500 kD are allowed to pass through this barrier, while molecules larger than 1000 kD cannot (Shalgi et al., 1973). As the densest and smallest lipoprotein, HDL is the only type of lipoprotein that is able to cross this barrier.

HDL remodeling is the structural re-arrangement of HDL mediated by cell receptors (Martinez et al., 2004), enzymes, and plasma proteins: ABCA1, ATP binding cassette subfamily G member 1 (ABCG1), and SCARB1 mediate the cholesterol efflux to extracellular acceptors; plasma CETP and phospholipid transfer protein (PLTP) mediate the transport of cholesterol and phospholipid among HDL, LDL, and VLDL; plasma lecithin, a cholesterol acyl transferase (LCAT), mediates the conversion of nonesterified cholesterol into esterified cholesterol (Fujimoto et al., 2010). These enzymes and protein transporters are also found in the follicular microenvironment. ApoA-1 content in follicular fluid can affect LCAT activity (Balestrieri et al., 2001). ABCA1 is expressed in mouse oocytes and mediates oocyte cholesterol efflux (Quiroz et al., 2020). Activated liver X receptors (LXRs) in human luteinized granulosa cells increase the cholesterol efflux by inducing the expression of the LXR target gene, *ABCA1*, *ABCG1*, *ApoE*, and *PLTP*, thus mediating cellular cholesterol homeostasis and hormone secretion (Drouineaud et al., 2007). Though follicles have been shown to affect lipoprotein synthesis and remodeling, studies from genetically modified mouse models showed that only *Scarb1* knockout mice had impaired fertility, while *Lcat*, *Abca1*, and *Apoa1* knockout mice procreate offsprings normally (Fujimoto et al., 2010), indicating that *Scarb1* plays a key role in maintaining female fertility *via* HDL remodeling (Table 1).

Recent studies have indicated the presence of other lipoprotein classes such as LDL and VLDL in follicular fluid, though their biosynthetic pathways remain unknown. Apart from ApoB, microsomal triglyceride transfer protein (MTTP), and ApoE, Apo-B100 was also found in the endoplasmic reticulum of granulosa cells from patients undergoing controlled ovarian hyperstimulation treatment (Gautier et al., 2010). The supplementary oleic acid in the IVM medium increased the production of ApoB100-containing lipoproteins (Gautier et al., 2010), while gonadotropin inhibited *ApoB* gene expression (Scalici et al., 2016). These findings indicate that LDL and VLDL in follicular fluid may come from granulosa cells. However, considering that ovulation induction drugs can affect lipid metabolic gene expression (Wang et al., 2015), whether or not granulosa cells in physiological conditions can produce and secrete lipoproteins remains to be further studied.

## Transcriptional Pattern of Lipid Metabolism Genes in Oocytes and Granulosa Cells During Folliculogenesis

**FIGURE 2:**
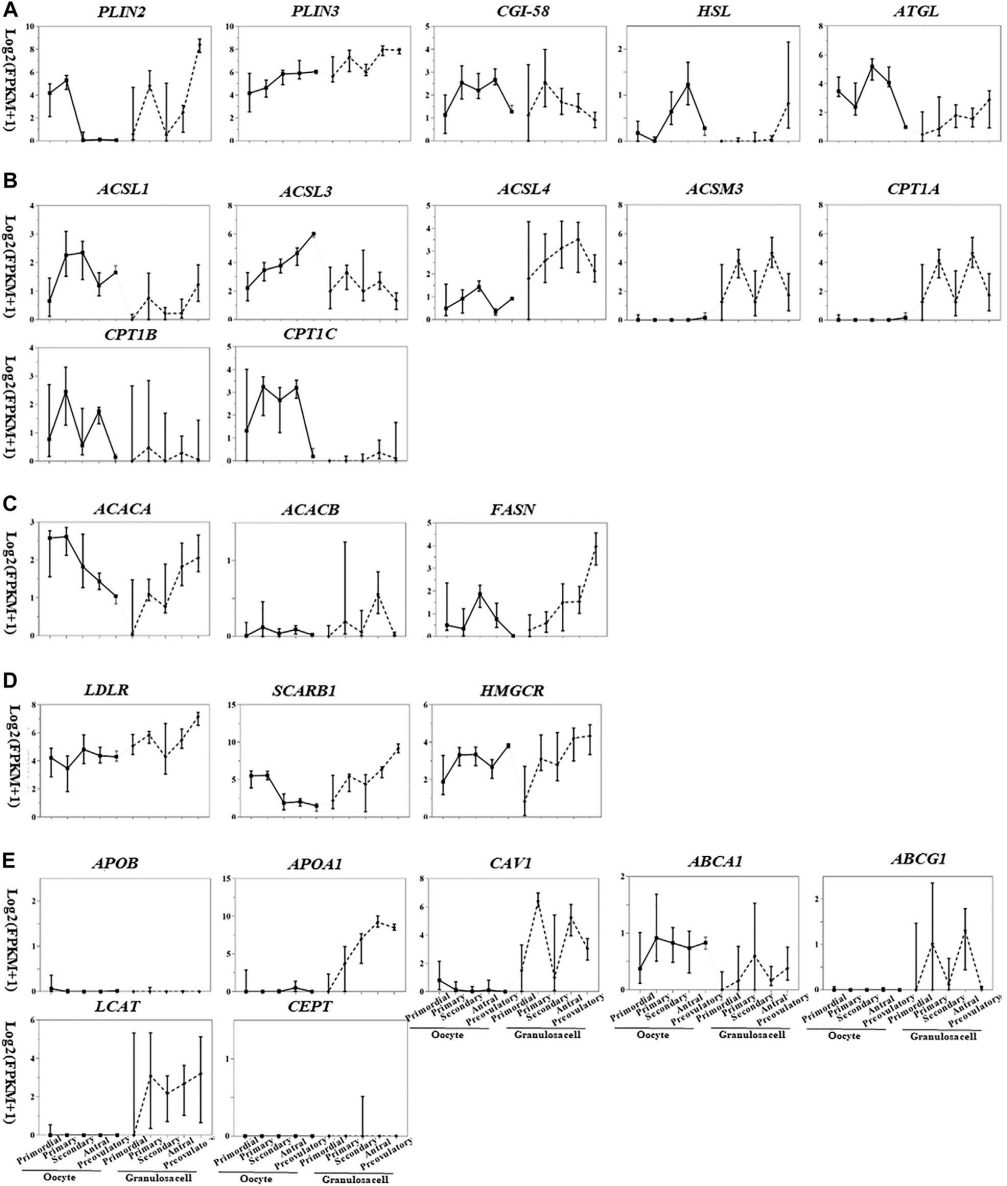
Transcriptional pattern of lipid metabolism genes in oocytes and granulosa cells during folliculogenesis [image sourced from (Martinez et al., 2004)]. **(A)** Triglyceride breakdown; **(B)** fatty acid oxidation; **(C)** fatty acid synthesis; **(D)** lipoprotein transport and cholesterol uptake; **(E)** lipoprotein synthesis and remodeling.

Based on the transcriptome database of follicular cells during folliculogenesis (Zhang et al., 2018), we generated expression maps of lipid metabolism factors in oocytes and granulosa cells at different stages (Figure 2), which partially reflect the cell- and stage-specific activity of lipid metabolism pathways in follicles. By comparing gene expression in antral and preovulatory follicles, potential lipid metabolism pathway functions in oocyte meiotic resumption and maturation can be hypothesized since the completion of MI occurs at preovulatory follicles.

The gene expression related to triglyceride breakdown is decreased in oocytes (e.g., *CGI-58*, *HSL*, and *ATGL*) and increased in granulosa cells (e.g., *PLIN2*, *HSL*, and *ATGL*) (Figure 2). *PLIN3* is highly expressed in both oocytes and granulosa cells among the three PLIN subtypes. *PLIN2* was highly expressed in granulosa cells of preovulatory follicles (Figure 2A), suggesting the potential role of PLIN2 in oocyte meiotic resumption.

Fatty acid degradation process includes the activation of fatty acids mediated by acyl-CoA synthetase (ACS), the entry of acyl-CoA into mitochondria mediated by CPT1 and CPT2, and β-oxidation mediated by acyl-CoA dehydrogenase (ACAD), enoyl-CoA hydratase (ECH), hydroxyacyl-CoA dehydrogenase (HADH), and ketoacyl-CoA thiolase (KAT). CPT1 is the rate-limiting enzyme for this process. According to the substrates and distribution sites, each enzyme is divided into several subtypes with different expression levels in oocytes and granulosa cells at different stages. Acyl-CoA synthetase long chain family member 1 (*ACSL*), *ASL3*, carnitine palmitoyltransferase 1B (*CPT1B*), and *CPT1C* are highly expressed in oocytes, and ACSL3, ACSL4, acyl-CoA synthetase medium chain family member 3 (ACSM3), and CPT1A are highly expressed in granulosa cells (Figure 2B). The expression of *CPT2*, *ACAD*, *ECH*, *HADH*, and *KAT* is not cell- or stage-specific (Figure 2B). During the transition from antral to preovulatory follicle, the expression of fatty acid activation–related genes is increased (*ACSL1, ACSL3, ACSL4*) or decreased (*ACSL3*, *ACSL4*, *ACSM3*) in oocytes and granulosa cells, while the expression of β-oxidation–related genes (oocytes: *CPT1B*, *CPT1C*; granulosa cells: *CPT1A*) was all decreased (Figure 2B). This decreased expression level of β-oxidation–related genes is inconsistent with the upregulated β-oxidation process as shown in previous studies (6). Metabolic enzymes synthesized during folliculogenesis after primordial follicle activation are inactive and stored in the oocytes, waiting for LH/hCG signals to be activated by covalent modification or zymogen activation, which may explain the contradiction.

Though whether oocytes can synthesize fatty acids is still controversial, *ACAC* (rate-limiting enzyme of fatty acid synthesis, including two subtypes *ACACA* and *ACACB*) and *FASN*, two enzymes required for fatty acid synthesis, are expressed at low levels in oocytes (Figure 2C), implying limited fatty acid synthesis in human oocytes.

*LDLR* and *SCARB1* are expressed in human oocyte and granulosa cells, especially in granulosa cells from secondary and preovulatory follicles (Figure 2D), providing sufficient substrates for energy supply and hormone synthesis. *ApoB* is neither expressed in oocytes nor in granulosa cells (Figure 2E). In addition to the weakly expressed gene *ABCA1*, genes related to HDL remodeling are not expressed in oocytes; however, these genes are all expressed in granulosa cells, and the expressions are especially high in antral follicles (Figure 2E), implying the crucial role of granulosa cells in HDL remodeling in the follicular microenvironment. Caveolin-1 (*CAV1*) is involved in SCARB1-mediated intracellular cholesterol efflux and is also expressed in granulosa cells (Figure 2E), suggesting that SCARB1 in granulosa cells may mediate the selective uptake and efflux of intracellular cholesterol and participate in the cholesterol homeostasis maintenance in the follicular microenvironment.

In conclusion, all lipid metabolic pathways can be carried out in oocytes and granulosa cells except for fatty acid synthesis, and the integrated effects of these pathways, including triglyceride breakdown, fatty acid oxidation, and cholesterol uptake in granulosa cells during meiotic resumption, are to provide energy supply and promote hormone synthesis. The expression of most genes related to lipid metabolism in oocytes is reduced during the transition from antral to preovulatory follicle, which may be induced by the general transcription inhibition in meiotic resumption. Further proteomic analysis of folliculogenesis will help understand the important role of lipid metabolism in meiosis and female reproduction, as the regulation of gene expression in meiotic resumption is mainly through translation and posttranslational modification rather than transcription (Susor et al., 2016; Luong et al., 2020).

## Effects of Lipid Metabolism on Oocyte Maturation

Lipid metabolism has a bidirectional effect on folliculogenesis and oocyte maturation. On the one hand, increased levels of some lipids are a protective factor for folliculogenesis due to the requirement of fatty acids in oocytes in energy-consuming events such as meiotic resumption and fertilization. For example, the number of oocytes retrieved and embryo formation are positively correlated with oleic acid levels in follicular fluid (Zarezadeh et al., 2020). Added melatonin in the IVM medium can improve the maturation rate, fertilization rate, and embryo formation rate (Jin et al., 2017; Li et al., 2019); furthermore, it can increase the lipid droplet content in porcine oocytes after maturation and upregulate the expression of genes related to lipogenesis, lipolysis, β-oxidation, and mitochondria biogenesis (*ACACA*, *FASN*, *PPARγ*, *SREBF1*, *ATGL*, *HSL*, and *PLIN2*) (Jin et al., 2017). Carnitine facilitates the transport of lipoyl CoA from the cytoplasm to mitochondria in fatty acid oxidation. L-carnitine added in the IVM medium increases follicular β-oxidation levels, thus improving the fertilization rate and blastocyst formation rate (Dunning et al., 2011). On the other hand, long-term exposure to a high-fat environment increases lipid levels in oocytes of women with obesity or polycystic ovary syndrome (PCOS) and induces lipotoxicity in oocytes, including the increase in oxidative stress and inflammation levels and disruption of spindles and chromosome structure, thus seriously interfering oocyte meiosis (Hou et al., 2016; Yun et al., 2019; Rao et al., 2020). Previous studies showed that the developmental potential of oocytes growing in follicular fluid with high content of stearic acid, palmitic acid, or oleic acid has decreased significantly (Valckx et al., 2014). Mouse COCs cultured in lipid-rich follicular fluid are found to have significantly decreased maturation rate and increased endoplasmic reticulum stress (Yang et al., 2012). The maturation rate, cleavage rate, and blastocyst rate of COCs treated with 50 μM linolenic acid are increased with upregulated levels of PGE2, cAMP, and MAPKs in oocytes. However, treatment of COCs with high linolenic acid (>100 μM) inhibited cumulus expansion and decreased oocyte maturation rate (Marei et al., 2009). The combination of multiple fatty acids in the IVM medium also affects oocyte quality. High oleic acid (425 μM) reduced the mitochondrial membrane potential in cumulus cells and oocytes, increased the reactive oxygen species (ROS) levels, and inhibited cumulus expansion, thus inducing apoptosis of cumulus cells, forming poor quality embryos with more fragmentation and lowering good quality embryo rate and blastocyst formation rate (Marei et al., 2017). 50 μM of linoleic acid could reverse the negative effects of high oleic acid content on cumulus cells and embryos, restore cumulus expansion, and increase the blastocyst formation rate to the level of the control group (Marei et al., 2017). The structure of fatty acids also affects oocyte quality. For example, linoleic acid (18:2 n-6) inhibited cumulus expansion, hindered oocyte maturation, and reduced the cleavage rate and blastocyst formation rate (Marei et al., 2010), whereas trans-10 and cis-12 conjugated linoleic acid (t10c12 CLA), a conjugated linoleic acid isomer that regulates blood lipids, increased oocyte maturation rate, cleavage rate, and blastocyst formation rate by upregulating the expression levels of phosphorylated MAPK3/1 and COX2 in COCs (Jia et al., 2014). Oocyte *in vitro* maturation conditions are controlled by the simulation of the microenvironment to regulate oocyte maturation. Therefore, previous *in vitro* experiments show that changes of lipids and related metabolic enzymes in the maternal environment and follicular cells have an impact on oocyte maturation and subsequent embryonic development, and the underlying mechanisms may include regulating the activity of factors of meiosis and oocyte maturation (PGE2, cAMP, and MAPKs), oxidative stress levels, and organelle structures and functions. However, the key regulatory factors involved in this process remain to be further studied.

FF-MAS (follicular fluid–meiosis-activating sterols) is derived from lanosterol by catalyst CYP51A1. As an intermediate in cholesterol biosynthesis, FF-MAS in follicular fluid is found to promote oocyte meiotic resumption (Byskov et al., 2002). FF-MAS added in the IVM medium of mouse COCs significantly increased oocyte maturation and embryo developmental potential (Marín Bivens et al., 2004a; Marín Bivens et al., 2004b; Guo et al., 2020). FSH (follicle stimulating hormone) stimulated FF-MAS secretion (Byskov et al., 1997) by increasing the expression of CYP15A1 (Nakamura et al., 2015). FF-MAS promoted meiotic resumption by the LXR receptor and MAPK/ERK signals in oocytes, but not the cAMP common in this process (Dallel et al., 2018; Guo et al., 2020). In addition, FF-MAS reduced the proportion of premature sister chromatid separation and aneuploidy rate in oocytes (Cukurcam et al., 2007). Though it is proven that FF-MAS can promote meiosis, whether it is necessary in meiotic resumption remains controversial. CYP51A1 inhibitors showed no inhibition on both *in vivo* and *in vitro* meiotic resumption (Tsafriri et al., 2002). The increase of FF-MAS in ovaries of mice treated with hCG occurred after germinal vesicle breakdown, which contradicted the hypothesis that FF-MAS could promote oocyte meiosis (Tsafriri et al., 2002; Tsafriri and Motola, 2007). Therefore, FF-MAS has the function of promoting oocyte meiosis through the MAPK/ERK signaling pathway, but it is not a key modulator in this process.

## Conclusion

Follicles are complex aggregations comprising germ cells and various somatic cells, and the regulation of folliculogenesis not only relies on the complex signal communication within follicles but also on the systemic functioning of the body. Lipid metabolism is one of the important biological events in the follicle microenvironment during folliculogenesis. In this review, we summarized the current research findings of the relationship between lipid metabolic pathways and meiosis in mammals. Fatty acid oxidation provides sufficient energy for meiotic resumption and lipid metabolites that participate in the regulation process. Lipoproteins, the main carriers of cholesterol and triglycerides in blood circulation, are involved in follicular cholesterol homeostasis and oocyte meiosis. Endogenous and exogenous signals such as gonadotropins, insulin, and cortisol act as upstream regulators to regulate follicular lipid metabolic homeostasis, thereby maintaining oocyte meiosis and folliculogenesis. These signal molecules are intertwined to form a complex regulatory network, but the key factors and main regulatory pathways that play a central role in this network still need to be further explored.

An important feature of folliculogenesis is that it is also the process of oocyte maturation. The quality of mature oocytes is closely associated with embryo development and the health of the offspring. Exploring the influence of lipid metabolism on folliculogenesis is helpful in understanding the mechanisms of how a maternal high-fat environment causes irreversible damage to the oocytes. The collective information summarized from the current and future studies will provide guidance for finding novel methods to protect ovarian reserves and improve ART outcomes in obese women, especially those with PCOS.
